# Liquid-liquid phase separation in cancer therapy resistance

**DOI:** 10.1016/j.isci.2026.115420

**Published:** 2026-03-20

**Authors:** Peng Hu, Huaze Ding, Ningning Yue, Jianxin Ye, Shengzhe Ruan, Yike Qian, Changping Wang, Dianwen Song

**Affiliations:** 1 School of Health Science and Engineering, University of Shanghai for Science and Technology, Shanghai 200093, China; 2 Department of Orthopedics, Shanghai General Hospital, Shanghai Jiao Tong University School of Medicine, Shanghai 200080, China; 3 Department of Gastroenterology, Shenzhen People’s Hospital (The First Affiliated Hospital, Southern University of Science and Technology, The Second Clinical Medical College, Jinan University), Shenzhen 518020, China

**Keywords:** cancer, molecular biology, oncology

## Abstract

Liquid-liquid phase separation (LLPS) has emerged as a fundamental mechanism that orchestrates essential cellular functions. Emerging evidence has established a compelling link between LLPS and core oncogenic processes, including cancer initiation, metastasis, immune evasion, and therapeutic resistance. Among these processes, treatment resistance remains a major barrier to achieving sustained clinical benefit. Aberrant biomolecular condensates formed via LLPS are increasingly recognized as critical mediators of multiple therapy resistance pathways and can be as promising therapeutic targets to overcome treatment failure. In this review, we synthesize emerging insights into how LLPS-derived biomolecular condensates contribute to therapy resistance through diverse mechanisms. We highlight therapeutic strategies aimed at disrupting or exploiting condensate dynamics for reversing cancer therapy resistance. By bridging phase separation biology with resistance mechanisms, this review offers a conceptual framework to assist future research and therapeutic development in oncology.

## Graphical abstract

**Figure:**
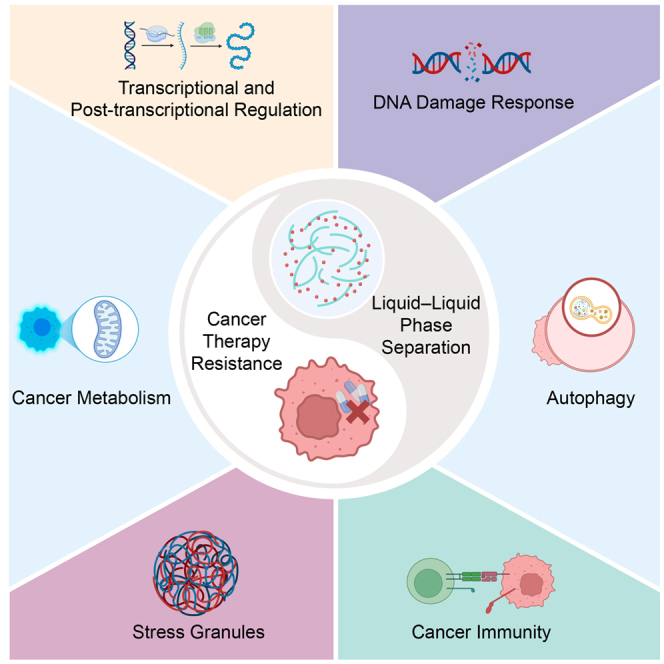

## Introduction

Phase separation refers to the phenomenon in which a uniform macromolecular solution undergoes a transition, resulting in the formation of two distinct phases: one enriched with the macromolecules and the other with a reduced concentration of these molecules.^1^ Intracellular biomolecular condensates are also referred to as membraneless organelles or droplets.^2^^,^^3^ Recent advances have established that these dynamic subcellular assemblies arise through liquid-liquid phase separation (LLPS), enabling cells to compartmentalize biochemical activities without the need for lipid membranes.^4^^,^^5^ Since the seminal discovery by Brangwynne et al., which demonstrated that P granules in *C. elegans* exhibit phase-separated behavior, the formation, localization, and function of biomolecular condensates have emerged as a major focus of cell biology.^6^^,^^7^^,^^8^ A wide array of biomolecular condensates has been identified, including representative condensates such as nucleoli, stress granules (SGs), Cajal bodies, and processing bodies, among others, each orchestrating specific cellular functions.^9^^,^^10^^,^^11^ Biomolecular condensates undergo dynamic assembly and disassembly, facilitating the spatial and temporal regulation of essential processes such as reaction kinetics, macromolecular organization, subcellular localization, and molecular concentration gradients.^12^^,^^13^^,^^14^^,^^15^

Importantly, aberrant condensate dynamics and dysregulated phase separation behavior have been increasingly implicated across a broad spectrum of human diseases, such as neurodegenerative disorders, cancer, and infectious diseases.^16^^,^^17^^,^^18^^,^^19^^,^^20^ Biomolecular condensates act as master organizers in cancer, orchestrating key oncogenic processes such as super-enhancer (SE)-driven transcription, signal transduction, and immune evasion.^20^^,^^21^^,^^22^ Functionally, they fuel proliferative and biosynthetic programs, couple the cell cycle to growth cues, and empower invasion and metastasis. In contrast, therapeutic challenge prompts cancer cells to rewire condensates into rapidly adaptable, cytoprotective hubs, which are reprogrammed to maintain survival by buffering drug-induced stress and blocking cell death pathways.^23^^,^^24^ Conceptually, whereas oncogenic condensates sustain tumor fitness and an aggressive phenotype, their therapy-resistant counterparts confer a dynamic, context-dependent survival advantage. Therefore, targeting biomolecular condensates offers a fresh perspective for overcoming resistance and devising precision therapeutics. This evolving field not only illuminates previously elusive mechanisms of tumor adaptability but also opens promising avenues for drug development against refractory cancers.

In this review, we summarize recent advances in understanding how LLPS contributes to cancer therapy resistance through diverse molecular mechanisms. We further highlight emerging therapeutic strategies that modulate condensate dynamics, offering promising opportunities to overcome treatment failure. By integrating mechanistic insights with clinical perspectives, this review aims to bridge LLPS biology and the pressing challenge of cancer therapy resistance.

### Search strategy and study selection

This narrative review was informed by a structured literature search to enhance transparency and reproducibility. PubMed and Web of Science Core Collection were searched from database inception to November 2025 using combinations of terms related to liquid-LLPS/biomolecular condensates (e.g., “liquid-liquid phase separation”, “LLPS”, “biomolecular condensate”, and “phase separation”), cancer (e.g., cancer, tumor/tumor, and neoplasm), therapy (e.g., therapy, treatment, drug, chemotherapy, radiotherapy, immunotherapy, and targeted), and resistance (e.g., resistance, drug resistance, therapy resistance, chemoresistance, and radioresistance). The full search strings for PubMed and Web of Science are provided in Table S1. In addition, Google Scholar was used as a supplementary source to identify relevant records using keyword combinations aligned with the above concepts and to support citation tracking of key papers. Titles/abstracts and, when necessary, full texts were screened for relevance to LLPS/condensate-associated mechanisms of resistance to chemotherapy, targeted therapy, radiotherapy, or immunotherapy. We prioritized original studies that provided mechanistic evidence linking condensates/LLPS to therapy resistance, while selected review articles were included for background synthesis and conceptual framing. Non-cancer studies and papers without a mechanistic linkage between condensates/LLPS and resistance phenotypes were excluded. A formal tool-based risk-of-bias assessment was not performed. Instead, to improve transparency in evidence interpretation, a qualitative evidence-strength appraisal was conducted for key primary studies supporting the major mechanistic claims. Studies were categorized into three evidence tiers based on the presence of orthogonal condensate/LLPS evidence and functional validation (perturbation/rescue and/or *in vivo*/clinical support), as summarized in Table S2.

### Biochemical and biophysical principles of LLPS in therapy resistance

**Figure 1:**
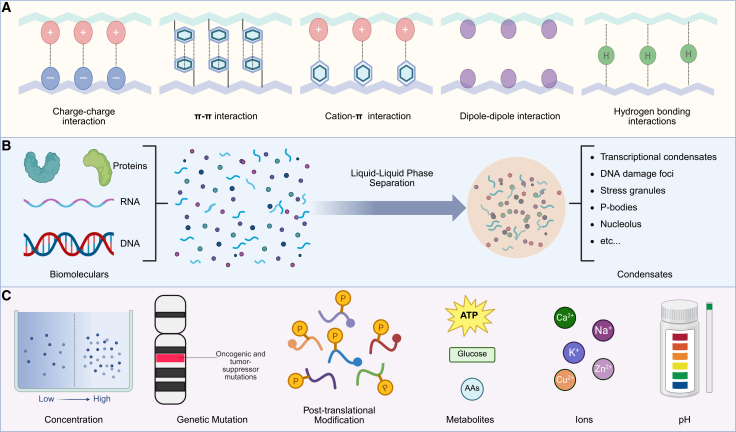
Biomolecular condensates formed via liquid-liquid phase separation (LLPS) (A) Molecular interactions underlying LLPS. (B) Key biomolecules, biological processes, and representative intracellular condensates. (C) Potential factors modulate LLPS in tumor cells. Created in https://BioRender.com.

LLPS is driven by multivalent, weak intermolecular interactions mediated by specific molecular features.^9^^,^^11^^,^^12^ Key contributors include intrinsically disordered regions (IDRs) and low-complexity domains enriched in aromatic, arginine, and lysine residues that enable π-π and cation-π interactions, while acidic residues and nucleic acids provide complementary electrostatic contacts.^25^^,^^26^^,^^27^ These interaction “stickers” separated by flexible “spacers” encode the phase behavior of biomolecules^1^^,^^28^ (Figures 1A and 1B).

In therapy-resistant cancers, these intrinsic sequence features are co-opted by dysregulated regulatory inputs. Oncogenic alterations, such as mutations, amplifications, and fusions in genes encoding coactivators, transcription factors (TFs), or DNA damage response (DDR) components, can increase the concentration or valency of phase-separating proteins, driving aberrant condensate formation that sustains oncogenic signaling and repair under therapeutic stress.^29^^,^^30^^,^^31^^,^^32^ The physicochemical tumor microenvironment further modulates LLPS. Metabolites and ions, including ATP, polyamines, divalent cations, and shifts in pH, can directly shift phase boundaries.^33^^,^^34^^,^^35^ Metabolic reprogramming also influences LLPS indirectly by supplying substrates for post-translational modifications (PTMs).^36^^,^^37^ PTMs such as phosphorylation, arginine methylation, lysine acetylation, and PARylation critically tune protein charge, valency, and interaction preferences, thereby regulating condensate assembly, disassembly, and material state (Figure 1C).

Ultimately, therapy resistance arises when LLPS-prone sequences converge with oncogenic and metabolic dysregulation, shifting the phase behavior of transcriptional, chromatin, and DDR networks toward stabilized, pathological condensates. The following sections discuss how these principles underpin specific resistance mechanisms and therapeutic opportunities.

### Biomolecular condensates contribute to regulating cancer therapy resistance via LLPS

Over the past decade, a growing body of research has identified a variety of biomolecular condensates that play pivotal roles in shaping tumor cell fate. These biomolecular condensates, formed through LLPS, have been closely linked to fundamental oncogenic processes, including tumor initiation, metastasis, and immune evasion.^38^^,^^39^^,^^40^^,^^41^^,^^42^ In this section, we focus on emerging mechanisms by which LLPS-driven condensates regulate cancer therapy sensitivity, including transcriptional and translational regulation, modulation of DDR, metabolic reprogramming, adaptive autophagy, SG dynamics, and immune modulation. Together, these findings reveal an additional regulatory layer underlying the multifaceted nature of therapy resistance in cancer. Representative LLPS-driven condensates that have been mechanistically linked to tumor therapy resistance are summarized in Table 1.

**Table 1:** Inducing factors and consequences of LLPS-driven cancer treatment resistance

Resistance Mechanism	Inducing Factors	LLPS-related Biomolecular Condensate	Mechanism of Action	Outcome/Results	Reference
Transcriptional Reprogramming	oncogenic mutations, hyperactivation of TFs	super-enhancers (FOXM1, BRD4 and others)	formation of transcriptional hubs to enhance gene expression	sustained tumor growth and resistance to chemotherapy	Xie et al.^43^
DNA Damage Response	DNA damage, oxidative stress	MRN complex and Others	DNA repair condensates concentrating DDR factors	efficient DNA repair, reduced sensitivity to DNA-damaging agents	Wang et al.^44^; Wang et al.^45^
Metabolic Reprogramming	altered nutrient availability, oncogene activation	metabolic enzymes	condensation of enzymes to modulate metabolic pathways	metabolic adaptation and survival under treatment stress	Liu et al.^46^
Autophagy and Stress Granules	therapeutic stress, ROS accumulation	p62/SQSTM1, NBR1	autophagy-related condensates enhance the degradation of toxic material	enhanced cell survival under cytotoxic stress	Huang et al.^47^; Redding and Grabocka^48^
Immune Modulation	immune checkpoint activation, inflammation	PD-L1 condensates, IRF1	LLPS-driven condensates modulating immune responses	immune evasion and resistance to immune checkpoint blockade	Yu et al.^49^; Wu et al.^50^

### Transcriptional and post-transcriptional regulation via LLPS

Gene transcription and translation are tightly regulated biological processes essential for maintaining cellular identity and function. Specifically, TFs, coactivators, chromatin remodelers, and RNA-binding proteins (RBPs) undergo LLPS to form biomolecular condensates at active chromatin sites, particularly at SE regions.^51^^,^^52^^,^^53^^,^^54^ These condensates can either activate or repress transcriptional programs, depending on their molecular composition and interaction networks.^55^ These phase-separated hubs exhibit hallmark properties such as dynamic fusion, liquidity, and selective permeability, which collectively enhance transcriptional robustness.^56^^,^^57^^,^^58^ In recent years, LLPS has been increasingly recognized as a pivotal mechanism for remodeling transcriptional programs during oncogenic transformation and therapy resistance.^59^

**Figure 2:**
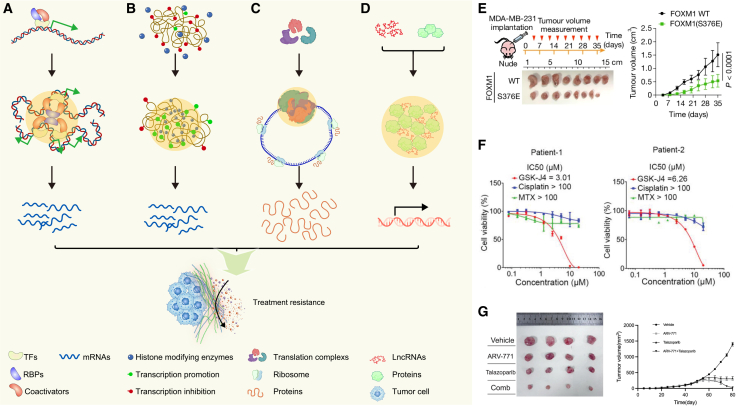
Transcriptional and post-transcriptional regulation via LLPS alters therapeutic sensitivity (A) Transcriptional addiction driven by the formation of enhancer or super-enhancer condensates comprising transcription factors (TFs), coactivators, and RNA-binding proteins (RBPs). (B) Aberrant biomolecular condensates formed by histone-modifying enzymes promote epigenetic remodeling within chromatin domains. (C) Biomolecular condensates formed by transcriptional initiators and cofactors enhance mRNA stability, contributing to post-transcriptional regulation. (D) Long non-coding RNAs (lncRNAs) facilitate the formation of pathological condensates, which in turn disrupt inhibitory signaling and trigger aberrant oncogenic activation. (E) Subcutaneous xenograft tumor growth of MDA-MB-231 cells expressing either wild-type (WT) Forkhead box protein M1 (FOXM1) or an LLPS-deficient mutant (S376E). (F) Cell viability assays of patient-derived primary cells resistant to methotrexate (MTX) and cisplatin, following treatment with MTX, cisplatin, or GSK-J4 (an inhibitor of HOXB8 phase separation). (G) Combinatorial efficacy of the FOXP1 condensate disruptor ARV-771 and Talazoparib in inhibiting tumor growth in patient-derived xenograft (PDX) models. The illustration of A-D was created in https://BioRender.com. The images of (E–G) were reproduced with permission from.^43^^,^^62^^,^^63^*Copyright © 2025 Springer Nature and 2021 John Wiley and Sons*.

Under therapeutic pressure, tumor cells dynamically rewire gene expression to maintain survival. LLPS facilitates this adaptation by enabling selective concentration and compartmentalization of transcriptional regulators.^54^^,^^60^^,^^61^ Figure 2 provides an overview of LLPS-driven transcriptional and translational condensates discussed in this section, highlighting how their context-specific composition and dynamics coordinate oncogenic gene-expression programs and contribute to therapy resistance.

#### Transcriptional reprogramming via LLPS

Under physiological conditions, LLPS-driven transcriptional condensates form at enhancers, promoters, and SEs to coordinate spatiotemporal gene expression programs and maintain cell identity. Under therapeutic or environmental stress, these same assemblies can be rewired to support adaptive, pro-survival transcriptional states.^60^^,^^64^

For example, Forkhead box protein M1 (FOXM1), a master regulator of cell proliferation, differentiation, and apoptosis, is tightly linked to malignant phenotypes in cancer.^65^ Xie et al. revealed that FOXM1 undergoes LLPS to form transcriptional condensates in breast cancer cell nuclei. These condensates are enriched with transcriptional coactivators such as Bromodomain-containing protein 4 (BRD4), Mediator Complex Subunit 1 (MED1), coalescing into SE-like hubs with high transcriptional activity.^43^ Functional validation in subcutaneous xenograft models demonstrated that disrupting FOXM1 phase separation significantly impairs tumor growth. At 35 days post-inoculation, the average volume of tumors expressing wild-type FOXM1 was approximately three times larger than those expressing an LLPS-deficient mutant (S376E), underscoring the necessity of FOXM1 condensation for tumor progression (Figure 2E). Similarly, core regulatory circuitry (CRC) composed of SE-associated TFs governs cell-type-specific gene expression programs, thereby determining cellular identity and function. Lu et al. identified HOXB8 and FOSL1 as CRC components associated with metastasis and therapy resistance in osteosarcoma. These factors were shown to undergo LLPS, forming biomolecular condensates that drive transcriptional addiction in cancer cells.^62^ Consistently, pharmacological interference with HOXB8 phase separation using GSK-J4 suppresses the growth of primary cell lines derived from patients resistant to cisplatin and methotrexate. These results highlight the potential of targeting LLPS-driven transcriptional networks to overcome multidrug resistance (Figure 2F).

In small cell lung cancer (SCLC), Tang et al. uncovered a pivotal role for LLPS-driven transcriptional condensates in orchestrating chemoresistance. The Forkhead box P1 (FOXP1) undergoes LLPS in response to cisplatin-induced stress, forming intranuclear condensates that recruit BRD4 and RNA polymerase II (RNAPII). These condensates amplify the transcription of DNA repair, anti-apoptotic, and interferon-response genes, collectively promoting a therapy-refractory state.^63^ The authors demonstrated that combining ARV-771 (a disruptor of FOXP1 condensates) with Talazoparib significantly enhances therapeutic efficacy in patient-derived xenograft (PDX) models (Figure 2G). This synergy highlights the potential of pharmacological condensate disruption in sensitizing resistant tumors to DNA-damaging agents. Similarly, retinoid X receptor γ (RXRγ) is uniquely overexpressed in chemo-resistant tumors and undergoes LLPS with lysine-specific histone demethylase 1A (LSD1) to form transcriptional condensates in SCLC. The process of LLPS enhances RXRγ-driven transcriptional activity, reprograms gene expression, and promotes tumor stemness and metastasis, ultimately contributing to chemo-resistance.^66^ Another study demonstrated that TRIM24 facilitates the assembly of fused in sarcoma (FUS) and glucocorticoid receptor (GR) into biomolecular condensates, which suppresses the transcriptional activity of GR. The condensates lead to diminished therapeutic efficacy of glucocorticoids and contribute to drug resistance in T cell lymphoma.^67^ Together, these findings highlight how LLPS-mediated remodeling of the transcriptional landscape under cytotoxic pressure can drive therapeutic resistance in cancer.

Castration-resistant prostate cancer (CRPC) exemplifies the role of LLPS in endocrine therapy resistance. Activated androgen receptor (AR) forms intranuclear condensates that enhance transcriptional output. While antiandrogens disrupt condensate formation of wild-type AR in sensitive tumors, mutant AR retains LLPS capacity even under treatment. Moreover, antiandrogens paradoxically promote condensate formation in the absence of ligand, sustaining downstream signaling and promoting resistance.^68^ In addition, Takayama et al. found that the TF complex (OCT4) in CRPC, aberrantly reactivated during prostate cancer progression, facilitates the assembly of subtype-specific transcriptional condensates in castration-resistant states.^69^^,^^70^ Thus, LLPS-mediated reprogramming of TFs circuitry enables phenotypic plasticity and therapeutic escape, further establishing biomolecular condensates as a critical driver of endocrine resistance in prostate cancer.

Yes-associated protein (YAP) functions as a transcriptional coactivator that orchestrates pro-survival and proliferative gene expression programs.^71^ Within the nucleus, YAP undergoes LLPS, forming dynamic biomolecular condensates that compartmentalize transcriptional components, including transcriptional coactivator with PDZ-binding motif (TAZ) and TEA domain transcription factor (TEAD), thereby amplifying transcriptional output from oncogenic loci.^72^^,^^73^^,^^74^ Aberrant activation of the YAP/TAZ-TEAD axis reconfigures the chromatin landscape, enabling cancer cells to evade cytotoxic pressure and persist under treatment stress.^75^^,^^76^ Torrino et al. found that extracellular matrix stiffening induces sorbitol accumulation through mechanosensitive metabolic reprogramming. Acting as an osmotic regulator, sorbitol fine-tunes the intracellular milieu to favor YAP phase separation, thereby facilitating the formation of transcriptionally competent condensates that support cellular persistence under drug-induced stress.^77^ Simultaneously, Haderk et al. revealed that the activation of focal adhesion kinase (FAK) promotes transcriptional activity of YAP, even though total YAP protein levels remain unchanged. During targeted therapy, a subset of cancer cells, termed drug-tolerant persister cells (DTPs), survive treatment and contribute to residual disease. FAK signaling in these cells promotes nuclear localization of YAP and likely stabilizes its biomolecular condensate structures by enhancing the interaction between YAP and TEAD, thereby sustaining transcriptional programs that enable therapeutic evasion.^78^

Beyond solid tumors, the oncogenic fusion protein NUP98-HOXA9 undergoes LLPS to form SE-like condensates in acute myeloid leukemia (AML).^79^^,^^80^ These condensates attract transcriptional coactivators and exclude repressors via molecular crowding, resulting in sustained oncogene activation and chemotherapy resistance. Phase-separated NUP98-HOXA9 also disrupts 3D genome architecture, reinforcing transcriptional dysregulation.^21^

Collectively, LLPS concentrates hub TFs and cofactors into enhancer/SE condensates, enabling rapid transcriptional rewiring toward pro-survival programs that promote tumor persistence and therapy resistance.

#### Epigenetic remodeling via LLPS

Physically, LLPS of chromatin-associated factors contributes to the spatial segregation of euchromatin and heterochromatin and to the local enrichment of epigenetic writers, erasers, and readers at specific genomic regions. When these condensates become deregulated in cancer, they can lock chromatin into oncogenic configurations that favor persistent transcription and treatment escape.

In multiple myeloma, steroid receptor coactivator-3 (SRC-3) forms condensates in cooperation with a histone methyltransferase (NSD2), increasing histone H3 lysine 36 dimethylation (H3K36me2) deposition at promoters of anti-apoptotic genes. This enhances survival signaling under proteasome inhibitor treatment. Inhibiting this interaction with SI-2 (SRC-3 inhibitor) restores sensitivity, illustrating the therapeutic relevance of disrupting condensate formation.^81^^,^^82^

Recent studies also highlight that chromatin-level phase separation enables topological reorganization required for sustained oncogenic transcription. In triple-negative breast cancer (TNBC), Lu et al. discovered that phosphorylated histone deacetylase 6 (HDAC6) undergoes LLPS to form nuclear condensates colocalized with core histones and heterochromatin markers.^83^ These phospho-HDAC6 condensates are not passive aggregates but serve as active chromatin organizers. They facilitate long-range chromatin looping and transitions between euchromatin and heterochromatin, thereby promoting pro-tumorigenic gene expression. Mechanistically, this LLPS behavior depends on Casein kinase 2-mediated phosphorylation at Ser22, and can be abrogated by site-specific mutation. These condensates are enriched at high-density chromatin loci marked by histone H3 lysine 9 trimethylation (H3K9me3) and heterochromatin protein 1α (HP1α), suggesting a scaffolding role in heterochromatin reprogramming. By reorganizing chromatin accessibility and enhancer-promoter interactions, HDAC6-mediated LLPS enables transcriptional reprogramming that promotes tumor progression and immune evasion. Targeting HDAC6 disrupts condensate formation, restoring normal chromatin architecture and attenuating malignant phenotypes.^84^ These findings establish phospho-HDAC6 condensates as a distinct class of LLPS regulators acting at the intersection of chromatin remodeling and transcriptional control. Together with TF-driven condensates (such as FOXM1 and YAP) and coactivator-dependent complexes (such as SRC-3/NSD2), HDAC6 condensates highlight the convergence of LLPS-mediated chromatin reprogramming in therapy resistance. However, the precise regulatory hierarchy and context-specific determinants of LLPS-mediated chromatin remodeling remain incompletely understood, warranting further mechanistic exploration.

Collectively, epigenetic condensates assembled by LLPS stabilize drug-tolerant transcriptional programs through two coupled layers: local enrichment of writers, erasers, and readers at specific loci and topological reorganization of chromatin compartments.

#### Condensate dynamics in post-transcriptional control

Under physiological conditions, LLPS underlies the assembly of diverse ribonucleoprotein granules, including SGs and P-bodies, which dynamically regulate mRNA stability, localization, and translation in response to environmental cues. Cancer cells hijack these post-transcriptional condensates to preferentially stabilize and translate pro-survival and pro-metastatic transcripts during therapeutic stress.^36^ RBP and N^6^-methyladenosine (m6A) readers can form phase-separated condensates that stabilize mRNA or regulate translation.^85^^,^^86^^,^^87^ In AML, YTHDC1 condensates maintain leukemic stemness by stabilizing m6A-modified transcripts. In oral cancer, circRNF13 enhances IGF2BP1 condensate formation, promoting ITGB1 mRNA stability and cisplatin resistance.^88^ These examples illustrate how RNA epigenetics and LLPS are closely intertwined in modulating therapeutic outcomes. Another study highlights poly(A)-binding protein cytoplasmic 1 (PABPC1) as a pivotal driver of blast crisis progression in chronic myeloid leukemia (CML) via LLPS. By assembling into condensates, PABPC1 selectively boosts the translation of leukemogenic transcripts enriched with long and highly structured 5′ untranslated regions (UTRs), thereby promoting disease aggressiveness and therapeutic resistance to tyrosine kinase inhibitors (TKIs).^89^

Overall, these findings indicate that LLPS contributes to therapy resistance not only through transcriptional rewiring but also via post-transcriptional control, particularly by modulating mRNA stability and translation through RBP- and RNA-driven condensates.

#### Long non-coding RNA-driven condensate dynamics

Physiologically, numerous long non-coding RNAs (ncRNA) act as architectural scaffolds that nucleate or remodel nuclear bodies and cytoplasmic ribonucleoprotein condensates through multivalent RNA-protein and RNA-RNA interactions.^90^ In cancer, aberrant expression or mutation of these long non-coding RNAs (lncRNAs) can reconfigure condensate composition and properties, adding an additional layer of LLPS-mediated control over gene expression and drug response. LncRNAs possess intrinsic structural features, such as extended secondary structures and RBP recognition motifs, that enable their incorporation into phase-separated condensates.^91^^,^^92^

LLPS-related lncRNAs such as ZNF32-AS2 are upregulated in colorectal cancer and exhibit a strong association with altered drug sensitivity profiles, particularly resistance to 5-fluorouracil. *In vitro* silencing of ZNF32-AS2 leads to enhanced apoptosis and suppressed colony formation in drug-treated cells. Bioinformatics integration reveals that LLPS-related lncRNA networks are enriched in chromatin remodeling pathways, RNA processing, and transcriptional regulation, suggesting that these RNAs influence condensate composition in a context-specific manner. The spatial localization and interaction propensity of lncRNAs appear to reconfigure the material properties of biomolecular condensates, modulating transcriptional output and potentially altering drug responsiveness.^93^ The precise mechanisms by which lncRNAs regulate transcriptional and translational processes via LLPS remain incompletely understood. Future investigations are warranted to elucidate how LLPS-associated lncRNAs modulate gene expression programs, especially in the context of disease states such as cancer.

In summary, LLPS emerges as a versatile and hierarchical mechanism that orchestrates gene regulatory programs across transcriptional, chromatin, and post-transcriptional layers. By spatially compartmentalizing molecular interactions, LLPS facilitates rapid and selective adaptation of tumor cells under pharmacological and environmental stress. These dynamic condensates support cellular plasticity, sustain oncogenic signaling, and promote therapeutic resistance. Despite growing insights into their biological significance, key mechanistic questions remain unresolved, particularly regarding the temporal dynamics of condensate formation, context-specific composition, and RNA or protein substrate selectivity. Elucidating these aspects may not only deepen our understanding of cancer pathophysiology but also inform the development of novel therapeutic strategies aimed at dismantling pathological phase-separated assemblies.

### Regulation of DNA damage response (DDR) via LLPS

**Figure 3:**
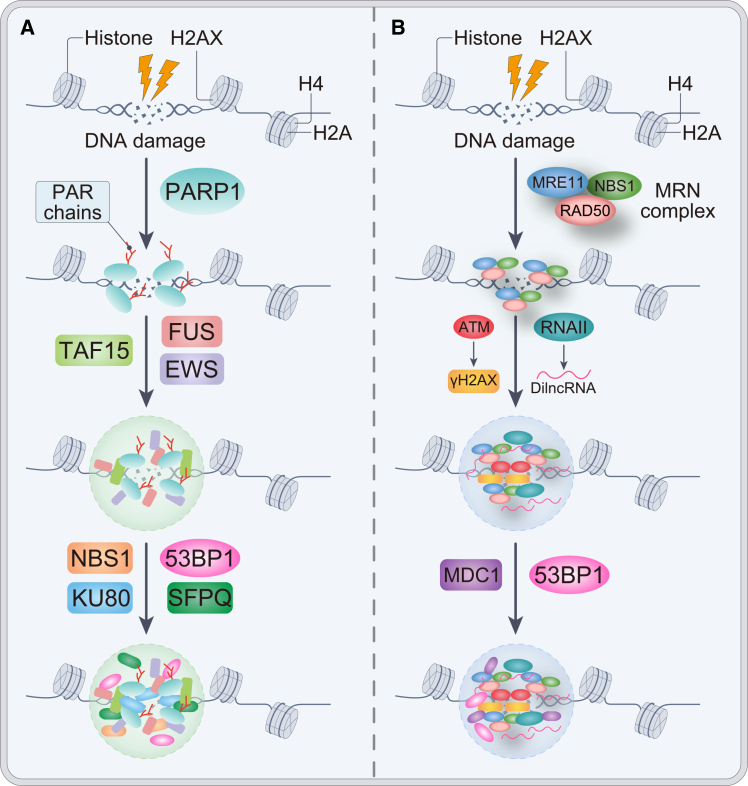
LLPS-mediated assembly of DNA damage response condensates at DNA double-strand breaks (A) Poly(ADP-ribose) polymerase 1 (PARP1) is rapidly activated at DNA damage sites and synthesizes poly(ADP-ribose) (PAR) chains on chromatin, which nucleate liquid-like condensates of the FET (FUS, EWS, and TAF15) family of RNA-binding proteins. These PAR-seeded droplets concentrate early DNA damage sensors and repair factors, including Nijmegen breakage syndrome 1 (NBS1), KU80, SFPQ, and p53-binding protein 1 (53BP1), thereby creating a permissive environment for efficient signal amplification. (B) The MRN complex (MRE11–RAD50–NBS1) recognizes DNA double-strand breaks and activates the kinase ataxia telangiectasia mutated (ATM), leading to histone H2AX phosphorylation (γH2AX). RNA polymerase II is then recruited to produce damage-induced long non-coding RNAs (lncRNAs), which, together with γH2AX and the adaptor mediator of DNA damage checkpoint 1 (MDC1), drive the formation of 53BP1-enriched repair condensates. Together, these PAR- and RNA-driven phase-separated compartments organize DDR signaling and repair.^96^ The illustration is adapted from an image by Ren et al. (https://doi.org/10.1002/advs.202202855), and licensed under the Creative Commons Attribution 4.0 International (CC BY 4.0, https://creativecommons.org/licenses/by/4.0/).

DDR is a fundamental mechanism by which cancer cells preserve genomic integrity and survive under genotoxic stress, including chemotherapy and radiotherapy. The efficiency and fidelity of DDR not only influence tumor aggressiveness but also determine the response to genome-targeting therapy.^94^^,^^95^ While traditional models emphasize enzyme-mediated signaling cascades and chromatin remodeling, emerging evidence reveals that biomolecular condensates formed via LLPS serve as a crucial regulatory layer in orchestrating the spatiotemporal dynamics of DDR.^44^^,^^96^^,^^97^^,^^98^^,^^99^ Figure 3 provides a schematic overview of how LLPS-driven condensates nucleated at DNA lesions spatially organize DDR factors and influence pathway choice.

These biomolecular condensates provide biochemical microenvironments that concentrate DDR factors, facilitate repair pathway assembly, and modulate the accessibility of damaged DNA.^100^^,^^101^^,^^102^^,^^103^^,^^104^ Importantly, aberrant or adaptive phase separation in tumor cells has been implicated in reprogramming DDR under therapeutic pressure, enabling evasion of cytotoxic effects and promoting resistance. In this section, we summarize current understanding of how LLPS regulates DDR and highlight key examples where phase separation actively influences repair pathway choice, repair kinetics, and ultimately, cancer therapy resistance.

Double-strand breaks (DSBs) represent one of the most deleterious forms of DNA damage and can be induced by commonly used chemotherapeutic agents such as doxorubicin and cisplatin. LLPS facilitates the spatial concentration of DDR-associated molecules at damage sites, forming repair condensates.^104^ The DNA break sensor complex MRE11–RAD50–NBS1 (MRN) is rapidly recruited to DSBs, where it initiates DDR signaling and promotes repair, thereby mitigating the genotoxic effects of chemotherapy and supporting tumor cell survival.^44^ Recent studies have shown that the MRN complex undergoes phase separation, with the IDR of Nijmegen breakage syndrome protein 1 (NBS1) modulating MRE11 ATPase activity and promoting MRN assembly and relocalization through dephosphorylation-dependent mechanisms, ultimately enhancing its accumulation at sites of DNA damage.^105^ Moreover, Zhang et al. demonstrated that the lactylation of MRE11 enhances MRN complex formation and activity, thereby promoting homologous recombination repair and attenuating chemotherapy-induced cytotoxicity.^106^ These findings suggest that PTMs may enhance the formation of MRN repair foci by LLPS.

In parallel, the MRN complex recruits RNAPII to initiate the transcription of damage-induced lncRNAs. These damage-induced lncRNAs can drive molecular crowding of DDR components such as 53BP1, facilitating the formation of DNA repair condensates and promoting the local enrichment of repair factors at DSB sites.^103^

Beyond its role in assembling DNA repair compartments, LLPS also modulates the dynamics of the DDR by regulating the availability of key repair factors. RNF168, an E3 ubiquitin ligase responsible for histone H2A ubiquitination, is a pivotal mediator of DSB repair, particularly within the non-homologous end joining (NHEJ) pathway. PTMs of RNF168 via SUMOylation induce its phase separation, leading to the formation of nuclear condensates that act as storage depots for the modified protein under basal conditions. Upon DNA damage, the SUMO-specific protease SENP1 is rapidly recruited to damage sites, where it catalyzes the deSUMOylation of RNF168. This enzymatic activity dissolves RNF168 condensates, thereby increasing the nuclear pool of free RNF168 available for DSB repair. The mobilization of RNF168 enhances NHEJ efficiency and has been implicated in promoting chemoresistance in cancer cells. Supporting this functional link, transcriptomic and genomic analyses reveal that SENP1 is frequently upregulated in colorectal cancer, where its expression correlates with poor clinical prognosis.^107^

Increasing evidence indicates that lncRNAs can undergo LLPS to form biomolecular condensates that spatially concentrate DNA repair factors at sites of damage.^97^ This compartmentalization facilitates the coordinated assembly of repair machinery, thereby enhancing DNA repair efficiency and promoting cellular resilience to genotoxic stress. For example, lncRNA-LINP1 has been reported to self-assemble into biomolecular condensates via RNA-RNA interactions, which serve as dynamic platforms to spatially concentrate and organize the DNA repair factors Ku70/Ku80. This facilitates Ku multimerization and end synapsis, thereby markedly enhancing the efficiency of NHEJ. Acting as a functional surrogate for the repair factor PAXX, LINP1 promotes DNA damage repair in response to chemo- and radiotherapeutic insults, ultimately contributing to therapy resistance in cancer cells.^108^^,^^109^

Beyond modulating the efficiency of DDR through various regulatory pathways, cancer cells can adaptively rewire DNA repair networks to overcome the effects of targeted inhibitors. This functional restoration of repair capacity, even in the presence of DNA repair-suppressive therapies, constitutes a key mechanism of acquired therapeutic resistance. Recent findings by Zhan et al. revealed that the histone acetyltransferase KAT6A promotes PARP inhibitor (PARPi) resistance in ovarian cancer through a non-enzymatic mechanism involving LLPS. In resistant cells, KAT6A forms nuclear condensates that sequester PARP1 away from DNA damage sites, thereby impairing PARP1 trapping, a key determinant of PARPi cytotoxicity, and enhancing homologous recombination repair. This condensate formation is driven by IDRs of KAT6A and stabilized by the scaffold protein APEX1.^110^ Notably, disrupting LLPS, rather than inhibiting KAT6A’s catalytic activity, was sufficient to restore PARPi sensitivity, highlighting the functional relevance of phase-separated condensates in modulating DNA repair and therapy resistance.

Cumulative evidence has established LLPS as a fundamental organizational principle in the regulation of DDR. By driving the formation of biomolecular condensates, LLPS enables the spatial and temporal coordination of repair signaling, factor recruitment, and repair pathway choice. This biophysical mechanism offers a level of regulatory flexibility that is particularly relevant under genotoxic stress, where rapid and localized control of DDR activity is essential for cell survival. Importantly, cancer cells can co-opt LLPS to maintain or restore repair capacity in the presence of therapeutic inhibitors, thereby contributing to adaptive resistance. These insights position LLPS not merely as a structural phenomenon but as a dynamic regulatory strategy that reshapes genome maintenance in malignant contexts. A deeper understanding of how condensate behavior influences DDR kinetics and repair fidelity may uncover new vulnerabilities exploitable for more durable cancer therapy responses.

### Regulation of cancer metabolism via LLPS

**Figure 4:**
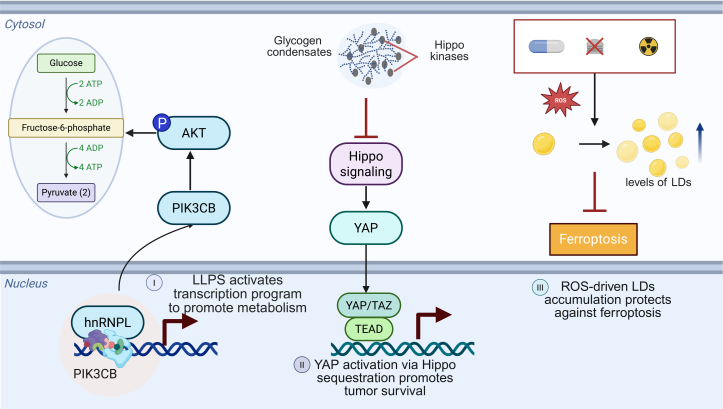
LLPS-mediated metabolic plasticity (I) Heterogeneous nuclear ribonucleoprotein L (hnRNPL) undergoes LLPS to form transcriptional condensates at the PIK3CB promoter. This enrichment enhances PIK3CB expression, subsequently activating the AKT signaling pathway to promote glycolysis. (II) Accumulated glycogen drives the formation of biomolecular condensates that sequester key Hippo kinases. This spatial partitioning inactivates the Hippo signaling pathway, thereby triggering the nuclear translocation of YAP to sustain tumor cell proliferation. (III) Therapeutic stressors, including drugs, radiation, and starvation, elevate reactive oxygen species (ROS) levels. Elevated ROS triggers the formation and accumulation of lipid droplets (LDs), which serve as an antioxidant buffer. This process protects tumor cells from ferroptosis and enhances metabolic adaptation to treatment. Created in https://BioRender.com.

Metabolic reprogramming has emerged as a hallmark of cancer, enabling tumor cells to adapt to dynamic microenvironmental cues and therapeutic pressures.^111^^,^^112^ While aerobic glycolysis and glutamine addiction have long been recognized, recent studies highlight that metabolic plasticity extends far beyond canonical pathways.^113^ Tumor cells exploit diverse nutrient sources, engage in lipid remodeling, and rewire redox homeostasis to sustain growth and resist cytotoxic agents. Importantly, resistance to therapies has been increasingly linked to the acquisition of distinct metabolic states.^112^^,^^114^^,^^115^ LLPS adds a new dimension to our understanding of how these metabolic adaptations are orchestrated (Figure 4).^116^^,^^117^ Through the formation of biomolecular condensates, LLPS allows for dynamic compartmentalization of metabolic enzymes, cofactors, and signaling intermediates.^22^ This spatial segregation enables cells to regulate the efficiency, localization, and timing of key metabolic reactions in response to intracellular and extracellular changes. Rather than merely reflecting underlying metabolic flux, these condensates serve as platforms that orchestrate cancer-specific metabolic phenotypes, particularly in the setting of therapeutic challenge.

Recent findings increasingly suggest that such adaptive metabolic states are coordinated at multiple levels: They are transcriptionally instructed by oncogenic and stress-responsive transcriptional programs, and are further physically organized via LLPS.^118^ Qin et al. demonstrated that LLPS of the RBP of heterogeneous nuclear ribonucleoprotein L (hnRNPL) facilitates PIK3CB transcription and glycolysis. In ovarian cancer cells, ncRNA transcribed from the PIK3CB promoter recruits hnRNPL and nucleates its condensation. This process activates PIK3CB expression and the downstream AKT signaling pathway, ultimately driving glycolysis and tumor progression (Figure 4). Mechanistically, LLPS modulates critical metabolic nodes that underlie resistance phenotypes. For example, Liu et al. demonstrated that excessive glycogen accumulation in hepatocytes undergoes phase separation, forming condensates that sequester Hippo pathway kinases and promote YAP-dependent tumorigenic transcription, thus linking metabolic substrate buildup with tumor initiation via LLPS (Figure 4).^46^ Similarly, SGs were shown to sequester mitochondrial mRNAs and antioxidant enzymes, thereby preserving NADPH pools and redox homeostasis during chemotherapy. These structures may support the persistence of drug-tolerant cell states by sustaining glutathione biosynthesis and reactive oxygen species (ROS) detoxification capacity.^119^

A more direct link between LLPS and therapy resistance emerges in the context of lipid metabolism. In KRAS-mutant pancreatic cancer, cells under chemotherapy pressure upregulate fatty acid oxidation (FAO) to maintain mitochondrial function and ATP supply. Recent evidence indicates that FAO enzymes such as CPT1A and ACSL3 exhibit clustering behavior facilitated by phase separation, especially under low-glucose or hypoxic conditions, allowing efficient β-oxidation compartmentalization. Disruption of these condensates sensitizes resistant cells to metabolic inhibition and ROS-induced death.^65^^,^^120^^,^^121^ Furthermore, Gao et al. identified a lncRNA, URB1-antisense RNA 1 (AS1), in sorafenib-resistant hepatocellular carcinoma (HCC) cells, whose elevated expression correlates with poor clinical outcomes. Subsequent mechanistic investigations revealed that URB1-AS1 promotes LLPS of ferritin, leading to a reduction in intracellular labile iron and thereby attenuating sorafenib-induced ferroptosis.^122^

Beyond protein condensates, phase separation principles fundamentally govern the biogenesis of lipid droplets (LDs). LDs (LDs) arise at the endoplasmic reticulum, where neutral lipids phase-separate into lenses that bud into the cytosol—a process orchestrated by membrane biophysics and the seipin assembly machinery.^123^^,^^124^^,^^125^ Under therapeutic pressure, LD biogenesis confers a survival advantage. By sequestering peroxidation-prone polyunsaturated fatty acids within a neutral core via diacylglycerol acyltransferase (DGAT)-mediated synthesis, LDs preserve membrane integrity and shield slow-cycling, drug-tolerant cells from ferroptosis (Figure 4).^126^^,^^127^ Beyond this protective role, LDs buffer against lipotoxic and oxidative stress and regulate fatty acid flux to mitochondria at organelle contact sites.^128^ These structures also modulate pharmacodynamics by altering the intracellular partitioning and bioavailability of lipophilic drugs.^129^ Crucially, such lipid remodeling extends to the tumor microenvironment: Extracellular vesicle-associated arachidonic acid can drive lipid accumulation in tumor-infiltrating neutrophils, forcing them into an immunosuppressive state that undermines chemo-immunotherapy efficacy in TNBC.^130^ Collectively, these results underscore a broad paradigm in which LLPS of metabolic regulation serves to buffer intrinsic stress and hostile microenvironmental conditions, ultimately securing cancer cell survival.

Altogether, LLPS functions as an active organizer of metabolic adaptation rather than a passive outcome of biochemical activity. By forming condensates that spatially orchestrate enzymes, cofactors, and intermediates, LLPS provides tumor cells with the rapid and localized control needed to maintain metabolic resilience under cytotoxic stress. Disrupting these metabolic condensates may therefore represent a novel therapeutic avenue.

### Regulation of autophagy via LLPS

**Figure 5:**
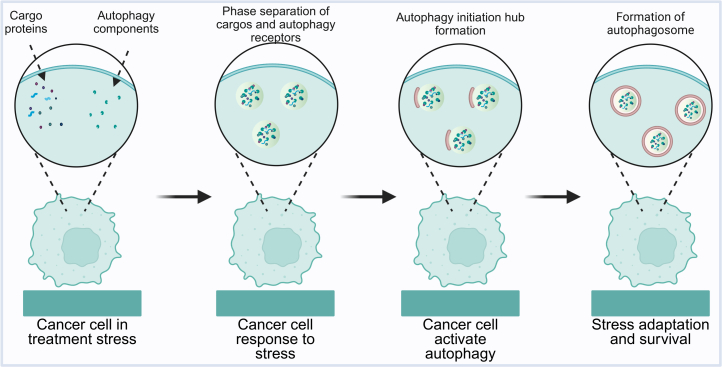
LLPS-mediated cargo recruitment and factor assembly during autophagy initiation LLPS facilitates the sequestration of autophagic cargo and the recruitment of key factors required for autophagy initiation. This coordinated assembly activates autophagy to sustain cell viability and homeostasis under environmental or therapeutic stress. Created in https://BioRender.com.

Autophagy, a conserved lysosome-dependent degradation process, plays a dual and context-dependent role in tumorigenesis and cancer therapy. While it suppresses early tumor development by eliminating damaged organelles and limiting genomic instability, it paradoxically facilitates cancer cell survival under metabolic, oxidative, and therapeutic stress by recycling macromolecules to sustain bioenergetic homeostasis.^131^^,^^132^ Recent insights have revealed that this functional plasticity of autophagy is not solely transcriptionally regulated but is orchestrated by LLPS, which compartmentalizes autophagic effectors, receptors, and cargo adaptors into dynamic, biomolecular condensates that modulate autophagic efficiency and selectivity (Figure 5).^133^^,^^134^^,^^135^^,^^136^^,^^137^

The autophagy receptor p62/SQSTM1 is a paradigmatic example of an LLPS-capable protein whose condensate behavior critically influences autophagy.^47^^,^^138^^,^^139^ Under stress conditions such as proteotoxicity or ROS elevation, p62/SQSTM1 undergoes LLPS via its PB1 domain and polyubiquitin-binding motifs, forming spherical condensates that act as pre-autophagosomal hubs to recruit ubiquitinated cargo and autophagy machinery, including LC3 and NBR1.^140^^,^^141^ This condensate formation not only ensures efficient autophagic clearance of toxic aggregates but also serves as a rheostat for selective autophagy activation.^142^ Wang et al. demonstrated that mutations disrupting p62/SQSTM1 phase separation impaired autophagosome recruitment and cargo degradation, indicating that LLPS is a prerequisite for the scaffolding function of p62/SQSTM1 in selective autophagy.^47^^,^^143^

More importantly, LLPS-modulated autophagy contributes to therapeutic resistance in cancer. In a study by Noguchi et al., exposure of cancer cells to TKIs induced lysosomal stress and enhanced LLPS of p62/NBR1, promoting the selective degradation of RhoA, a GTPase involved in cell motility. This mechanism highlights how LLPS-autophagy dynamics can determine treatment outcomes under drug exposure.^131^ In other settings, LLPS-enhanced autophagic flux may facilitate degradation of chemotherapy-induced damaged mitochondria (mitophagy), protecting tumor cells from apoptosis.^139^ Recently, Chen et al. revealed that the tyrosine kinase PTK6 promotes autophagy through an LLPS-dependent mechanism involving HNRNPH1. PTK6 phosphorylates HNRNPH1 at tyrosine-210, inducing its condensation into LLPS-driven droplets. These condensates alter HNRNPH1-mediated splicing of the autophagy receptor NBR1, specifically facilitating exon 10 inclusion and upregulating NBR1 expression, thereby enhancing autophagic flux and bolstering tumor cell survival under stress.^144^

Emerging data also point toward LLPS in the regulation of autophagy-mediated immune evasion. Tumor cells evade immunosurveillance in part by stabilizing immune checkpoint molecules such as PD-L1. It has been suggested that LLPS may regulate the turnover of such proteins via autophagy pathways, influencing immunotherapy response, although mechanistic details remain under investigation.^134^^,^^135^^,^^145^

Collectively, these findings support a model in which LLPS governs the spatial and temporal dynamics of autophagy to endow cancer cells with stress tolerance and therapeutic adaptability. Phase-separated autophagic condensates enable the formation of efficient degradation platforms that are not hardwired by genetic mutations but instead dynamically assembled in response to environmental challenges. The link between LLPS and autophagy is an attractive target for pharmacological intervention. Dissolution of aberrant condensates or interference with scaffold protein interactions may re-sensitize tumors to chemotherapeutics, targeted agents, and immunotherapies.

### Regulation of SGs and nuclear condensates via LLPS

**Figure 6:**
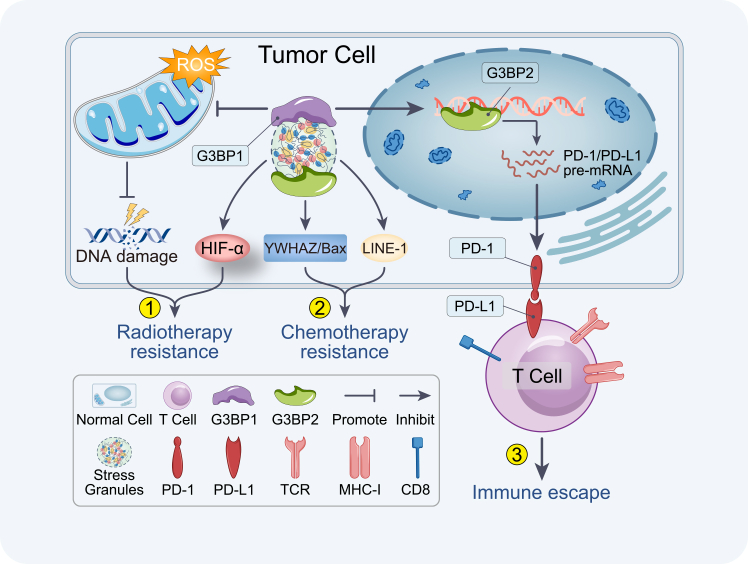
Stress granules (SGs) promote therapy resistance SGs facilitate radiation resistance, chemotherapy resistance, and promote immune escape, impairing the efficacy of immune checkpoint blockade (ICB) therapy.^157^ The illustration is adapted from an image by Zhou et al. (https://link.springer.com/article/10.1186/s13578-023-01030-6), and licensed under the Creative Commons Attribution 4.0 International (CC BY 4.0, https://creativecommons.org/licenses/by/4.0/).

SGs are formed via LLPS when eukaryotic cells encounter stress conditions such as translational arrest, proteotoxicity, oxidative stress, endoplasmic reticulum stress, or hypoxia.^146^^,^^147^ SGs assemble rapidly through the reversible aggregation of mRNAs and RBPs, enabling cells to fine-tune gene expression and signaling pathways in response to adverse stimuli.^148^^,^^149^^,^^150^^,^^151^^,^^152^ This ancient survival mechanism is co-opted to help tumor cells withstand therapeutic pressures. Facing interventions such as chemotherapy, radiotherapy, and immunotherapy, which induce DNA damage, oxidative stress, and apoptotic signaling, SG assembly is markedly enhanced in multiple tumor cell types.^153^^,^^154^ SGs sequester mRNAs encoding pro-apoptotic factors and key components of death-associated signaling pathways, slow global protein synthesis, and preserve critical survival transcripts and proteins.^121^^,^^155^^,^^156^ As a result, they contribute directly to therapy resistance by buffering tumor cells against the lethal effects of treatment (Figure 6).^48^^,^^158^^,^^159^

Specifically, exposure to chemotherapeutic agents such as cisplatin and oxaliplatin triggers SGs assembly through mechanisms involving eukaryotic initiation factor 2α (eIF2α) phosphorylation and interactions between intrinsically disordered protein regions. These SGs sequester pro-apoptotic factors such as Bax and RACK1, delaying cell death.^157^ Knockdown of G3BP1 increases sensitivity to both chemotherapy and radiation in several tumor types, highlighting its central role in protective SG formation.^160^ For example, the loss of G3BP1 in pancreatic ductal adenocarcinoma compromises resistance to oxidative stress, further demonstrating the importance of SGs in therapeutic resilience.^120^^,^^153^^,^^161^ Kim et al. established a new pathway (RNF144A-VRK2-G3BP1 axis), which regulates SG formation in osteosarcoma and links SG dynamics directly to chemotherapy resistance.^162^ Additionally, SGs have been shown to disrupt pro-apoptotic signaling pathways such as the MAPK and p53 pathway, reinforcing their function in chemoradio-protection.^48^^,^^154^ Recently, Meng et al. uncovered a critical link between SG dynamics and metabolic reprogramming in HCC. They demonstrated that the atypical kinase RIOK1 becomes enriched in SGs together with IGF2BP1 and G3BP1 under TKI treatment. Within biomolecular condensates, PTEN mRNA is sequestered. This downregulation of PTEN shifts metabolism toward the pentose phosphate pathway (PPP), increasing NADPH production and bolstering antioxidant capacity, thereby enhancing cell survival during TKI exposure. Notably, SGs marked by RIOK1 are present in donafenib-resistant HCC tumors from patients.^119^^,^^163^ This study further elucidates the crosstalk between SGs and metabolic reprogramming in cancer, deepening our understanding of the stress-adaptation responses utilized by tumor cells under therapeutic stress.

Together, these findings underscore that SGs act as multifaceted regulatory hubs that protect tumor cells from diverse therapeutic challenges by reprogramming RNA translation, sequestering pro-apoptotic signals, buffering intracellular stress, and modulating key signaling pathways.

Beyond cytoplasmic SGs, nuclear condensates, including the nucleolus and Cajal bodies, also remodel gene expression under therapy stress by acting upstream in the protein biogenesis process. The nucleolus serves as a key biomolecular condensate that regulates rRNA transcription and ribosome assembly. Therapeutic insults can disrupt this machinery and elicit nucleolar stress, fundamentally altering the organelle’s biophysical properties.^164^ This remodeling redirects protein synthesis toward adaptive survival programs, such as p53-dependent responses, thereby fostering drug tolerance rather than simple growth arrest.^165^^,^^166^^,^^167^ Similarly, Cajal bodies function as condensates that concentrate factors essential for small nuclear ribonucleoproteins maturation, spliceosome assembly, and telomerase biogenesis. Under therapeutic stress, these structures actively shape transcript diversity and RNA processing kinetics to support adaptation.^168^^,^^169^ In colon cancer, the phosphorylation of the Cajal bodies scaffold protein coilin by the kinase UHMK1 serves as a critical regulatory axis. This phosphorylation event modulates the dynamics of Cajal bodies assembly and disassembly in response to 5-fluorouracil treatment. Such remodeling triggers widespread shifts in alternative splicing profiles that enhance tumor cell survival and facilitate the transmission of adaptive signals within the tumor microenvironment. Consequently, Cajal bodies morphology and the phosphorylation status of coilin may serve as sensitive visual indicators of a cellular transition toward a therapy-resistant phenotype.^170^

Collectively, LLPS is related to a multi-compartment defense strategy. By coupling the sequestration of pro-apoptotic factors in cytoplasmic SGs with the adaptive remodeling of nuclear biosynthetic hubs, LLPS orchestrates a synchronized, coherent defense against therapeutic insults. This coordinated response allows cells to shut down bulk protein synthesis to conserve energy, while maintaining the translation of specific mRNAs required for survival. Consequently, this selective control helps drug-tolerant cells withstand prolonged treatment.

### Regulation of cancer immunity via LLPS

The interplay between cancer and the immune system is profoundly influenced by dynamic molecular compartmentalization within cells. LLPS is now recognized as a pivotal modulator of immune signaling fidelity and duration.^16^ By concentrating or sequestering key immune regulators, LLPS fine-tunes immune surveillance and contributes to both immunogenicity and immune escape mechanisms in cancer.^171^^,^^172^^,^^173^

Recent studies have illuminated how LLPS controls transcriptional responses to inflammatory cues. For instance, under interferon-γ (IFN-γ) stimulation, the lysine acetyltransferase KAT8 coalesces with IRF1 into transcriptionally active condensates, promoting robust PD-L1 expression and dampening T cell-mediated cytotoxicity.^50^ Pharmacological disruption of this condensate architecture has been shown to sensitize tumors to immune checkpoint blockade, unveiling LLPS as a tractable node to reverse immune resistance. In a related mechanism, IFN-γ also induces LLPS of YAP, which recruits TEAD4, EP300, and MED1 into nuclear condensates. These transcriptional hubs upregulate immunosuppressive genes, facilitating immune evasion. Interestingly, the study also noted that the formation of YAP condensates exhibited spatiotemporal specificity. IFN-γ remains at relatively low levels, and YAP stays in a dispersed state during the initial phase of treatment. As immune pressure escalates and the IFN-γ level surpasses a critical threshold, the number of YAP condensates progressively increases, consequently driving a transition in the immune phenotype from sensitivity to resistance against immunotherapy.^49^^,^^174^

Moreover, LLPS also interfaces with innate immunity by modulating the spatial availability of cytosolic sensors and downstream effectors.^175^ Tumors harboring *NF2* mutations exhibit pathological condensate formation involving IRF3, which impairs cGAS-STING axis activation by preventing TBK1 engagement. This spatial reconfiguration blunts type I interferon and fosters an immunologically “cold” tumor milieu, thereby facilitating the evasion of host immunity.^176^^,^^177^

Furthermore, pan-cancer transcriptomic analyses have identified LLPS-associated transcriptional programs linked to distinct immune phenotypes. In head and neck squamous cell carcinoma, LLPS-enriched subtypes exhibit a heightened expression of immune checkpoints and enhanced infiltration of effector lymphocytes—features predictive of improved responses to immunotherapies. These findings underscore the prognostic relevance of LLPS-driven transcriptional states in stratifying patients for immunotherapy.^178^^,^^179^ However, more detailed experimental evidence is required to robustly support this conclusion and to guide future immunotherapeutic strategies.

Taken together, LLPS emerges as a central regulator of tumor-immune interactions, not merely by orchestrating intracellular signaling but also by reprogramming immune contexture at the tissue level. Understanding and targeting LLPS-mediated immunomodulation may provide a novel avenue to overcome resistance to current immunotherapies and potentiate durable antitumor responses.

### LLPS-based therapeutic strategies to overcome tumor treatment resistance

**Figure 7:**
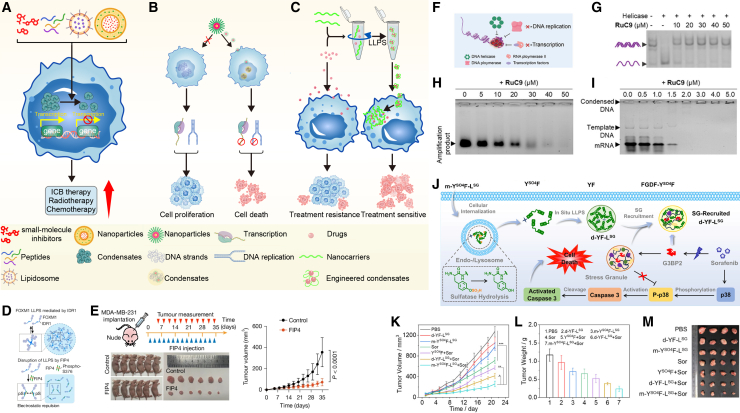
LLPS-based therapeutic strategies to overcome tumor treatment resistance (A) Disruption of specific intracellular biomolecular condensates through natural small-molecule inhibitors, synthetic peptides, or targeted delivery of inhibitory factors to sensitize tumor cells to conventional therapies. (B) Intracellular delivery of LLPS-inducing agents to trigger aberrant LLPS, thereby impairing therapeutic resistance or directly eliminating tumor cells. (C) *In vitro* engineering of artificial biomolecular condensates via LLPS to increase intracellular drug concentrations and enhance therapeutic efficacy. (D) Pharmacological inhibition of FOXM1 phase separation by the small-molecule inhibitor FIP4. (E) *In vivo* tumor suppression in MDA-MB-231 subcutaneous xenograft models following treatment with FIP4. (F–I) RuC9-mediated DNA condensation and its inhibitory effects on genomic processes. (F) Agarose gel electrophoresis demonstrating that RuC9-induced DNA condensation inhibits (G) DNA unwinding, (H) DNA replication, and (I) RNA transcription. (J) Schematic representation of *in situ* peptide phase separation. Peptides assemble into coacervate droplets within living cells, targeting SGs and thereby sensitizing tumor cells to sorafenib chemotherapy. (K–M) *In vivo* therapeutic evaluation of peptide-based condensates. (K) Tumor growth curves, (L) tumor weights, and (M) representative images of tumors dissected from mice following treatment with PBS, peptide formulations (d-YF-LSG, *m*-YSO4F-LSG), sorafenib (Sor), or indicated combinations. The illustration of A-C was created in https://BioRender.com. The images of D-M were reproduced with permission from.^43^^,^^183^^,^^184^*Copyright © 2025 Springer Nature and 2025 John Wiley and Sons.*

Recent advances have illustrated that LLPS is not merely a bystander but a key driver of therapeutic resistance, offering novel intervention points.^37^^,^^180^^,^^181^^,^^182^ Several case studies have demonstrated the potential therapeutic value of targeting LLPS to enhance drug sensitivity. A conceptual overview of LLPS-based therapeutic strategies is presented in Figure 7, and representative agents, molecular targets, and tumor contexts are summarized in Table 2. Later in discussion, we discuss three emerging approaches: disruption of pathological condensates, induction of aberrant condensates, and engineering of artificial condensates for drug delivery and therapeutic agents.

**Table 2:** Therapeutic strategies targeting biomolecular condensates in cancer therapy resistance

Resistance mechanism	Biomolecular condensate	Therapeutic strategy	LLPS-related mechanism of action	Evidence status	Reference
Transcriptional/epigenetic reprogramming	HDAC6 chromatin condensates	HDAC6-selective inhibitor nexturastat A	disrupts phospho-HDAC6 LLPS by blocking importin-β/14-3-3θ interactions, restoring chromatin architecture and transcriptional responses	experimental	Lu et al.^83^
Transcriptional/epigenetic reprogramming	FOXM1 transcriptional condensates	FOXM1-interfering peptides	reduce FOXM1 LLPS and suppress FOXM1 activity and target gene expression	experimental	Xie et al.^43^
Transcriptional/epigenetic reprogramming	FOXP1 super-enhancer condensates (SP8–HR axis)	PARP inhibitor+BET inhibitor	attenuate FOXP1-driven HR gene transcription and overcome FOXP1^ˆhigh^ chemoresistance	conceptual	Tang et al.^63^
Transcriptional plasticity/lineage reprogramming	OCT4–androgen receptor transcriptional condensates	nucleoside analogue ribavirin	disrupt OCT4 condensates and the OCT4–AR complex, limiting lineage plasticity and AR signaling	experimental	Takayama et al.^69^
DDR	MRN (MRE11–RAD50–NBS1) repair condensates	LDHA inhibitors (e.g., stiripentol)	reduce lactate production and NBS1/MRE11 lactylation, destabilizing MRN condensates and decreasing HR repair foci	experimental	Chen et al.^106^
DDR reprogramming (PARP1-dependent repair)	FUS-PAR DNA damage condensates	PARP1 inhibitors	block PAR synthesis is required for FUS recruitment and condensate formation at DNA damage sites, impairing DDR	experimental	Singatulina et al.^185^
DDR reprogramming (NHEJ pathway)	SUMOylated RNF168 nuclear condensates	SENP1 inhibition/modulation of RNF168 SUMOylation	maintain RNF168 LLPS and sequestration of 53BP1 to limit efficient NHEJ and sensitize cells to DNA-damaging therapy	conceptual	Wei et al.^107^; Feng et al.^186^
Metabolic reprogramming/Metabolic plasticity	metabolic enzyme condensates (e.g., glycolytic enzyme assemblies)	engineered peptides or small molecules targeting the enzyme LLPS	hypothetically disperse metabolic condensates to restrict therapy-induced metabolic adaptation	conceptual (no direct data)	Liu et al.^46^; Qin et al.^118^; Zhong and Yin^119^
Autophagy-dependent survival	p62/SQSTM1–NBR1 autophagy condensates	p62-targeting inhibitors or peptides	disrupt selective autophagy condensates, shifting the balance toward cell death and reducing therapy resistance	conceptual	Noguchi et al.^131^
Stress-response and drug-tolerant persisters	RIOK1–IGF2BP1–G3BP1 Stress granules	HDAC inhibitor chidamide	downregulates RIOK1, prevents RIOK1-positive stress granule formation, restores PTEN translation and enhances TKI efficacy	experimental	Meng et al.^163^
Immune evasion via PD-L1 upregulation	KAT8–IRF1 transcriptional condensates	2142–R8 blocking peptide	disrupts KAT8–IRF1 condensates, reduces PD-L1 expression and promotes antitumor immunity	experimental	Wu et al.^50^

### Disruption of pathological condensates

Disrupting specific intracellular biomolecular condensates through natural small-molecule inhibitors, synthetic peptides, or targeted delivery of inhibitory factors can resensitize tumor cells to conventional therapies (Figure 7A).^187^ For example, in TNBC, the phosphorylation-dependent phase separation of HDAC6 leads to the formation of nuclear condensates that promote chromatin reprogramming and oncogenic transcription. Targeting these condensates with small-molecule inhibitors has been shown to resensitize resistant tumor cells, providing a promising therapeutic strategy.^83^ Similarly, synthetic peptides targeting FOXP1-dependent transcriptional hubs can selectively dismantle these condensates, restoring chemosensitivity in SCLC.^63^ Xie et al. demonstrated that the inhibitor FIP4 suppresses FOXM1 phase separation, leading to significant growth inhibition of MDA-MB-231 tumors *in vivo* (Figures 7D and 7E). These findings confirm that the disruption of phase-separated transcriptional hubs represents a viable and potent therapeutic approach to overcome treatment resistance.

### Induction of aberrant condensates

Intracellular delivery of LLPS-inducing agents can trigger aberrant condensate formation, impairing resistance mechanisms or directly killing tumor cells (Figure 7B).^183^^,^^188^ For instance, the induction of phase-separated chromatin and DDR-related condensates has been identified as a potential therapeutic target in several cancers. In osteosarcoma, inhibition of H3K27 demethylase (GSK-J4) interferes with condensate formation in regulatory complexes, reversing transcriptional addiction and overcoming drug resistance.^37^ Similarly, disrupting SENP1-driven RNF168 condensates in colorectal cancer impairs DNA repair and enhances sensitivity to genotoxic therapies.^107^ Zhou et al. developed Ru(II) complexes (RuC9) that induce targeted DNA condensation. This localized phase transition exacerbates DNA damage and interferes with both replication and transcription, ultimately triggering apoptosis (Figures 7F–7I). Consequently, the induction of non-physiological phase separation to disrupt essential cellular functions represents a promising alternative strategy to circumvent therapeutic resistance.

### Engineering artificial condensates for cancer therapy

Beyond targeting endogenous condensates, the *de novo* engineering of artificial condensates represents a distinct strategy for cancer intervention (Figure 7C).^189^^,^^190^^,^^191^^,^^192^ This approach operates primarily through two mechanisms: serving as smart drug carriers or functioning as intrinsic therapeutic modalities.

First, artificial condensates can be engineered *in vitro* to serve as dynamic reservoirs that enhance drug retention and circumvent resistance. Unlike conventional rigid nanocarriers, phase-separated droplets provide high encapsulation efficiency for diverse payloads. For instance, peptide-drug conjugates (PDCs) can assemble into membraneless carriers that sequester chemotherapeutics such as doxorubicin within tumor cells, effectively evading efflux pump-mediated clearance.^188^ Beyond passive storage, functionalized condensates can modulate specific signaling nodes. For example, LLPS-mediated Wnt pathway inhibitors demonstrate efficacy in EGFR-TKI-resistant non-SCLC, while GalNAc-conjugated siRNA condensates restore sorafenib sensitivity in hepatocellular carcinoma by sustaining local gene silencing.^122^^,^^193^

Second, the *in situ* phase separation of synthetic molecules functions as a direct therapeutic modality.^191^ In this strategy, small molecules or peptides are designed to undergo supramolecular assembly specifically within the tumor microenvironment, triggered by cues such as overexpressed enzymes or ion gradients.^194^ These *in situ-formed* structures exert therapeutic effects through biophysical mechanisms rather than canonical ligand-receptor binding. Recent studies demonstrate that such assemblies can physically compromise organelle integrity or spatially sequester pro-survival proteins, thereby inducing cell death independent of traditional resistance pathways.^184^^,^^195^^,^^196^ Wang et al. developed an enzyme-triggered *in situ* phase separation system where peptides assemble into coacervate droplets. This strategy selectively induces condensate formation at SGs within tumor cells (Figure 7J). When combined with sorafenib, these in situ-formed droplets exhibit potent synergy, significantly suppressing tumor growth in A549 cell-derived xenograft models (Figures 7K–7M).^184^ In addition, Sain et al. introduced a non-peptide small molecule, DN6, that leverages tumor-specific nitroreductase (NTR) to initiate a liquid-to-solid phase transition (LST). Upon enzymatic reduction in hypoxic environments, DN6 undergoes *in situ* LLPS followed by maturation into cytotoxic solid nanoaggregates within mitochondria. These phase-separated structures disrupt mitochondrial integrity, leading to elevated ROS and triggering apoptosis independent of external stimuli.^197^ This underscores the potential of enzyme-instructed self-assembly to generate irreversible biophysical damage to specific organelles.

Collectively, these findings illustrate that LLPS can be therapeutically modulated along three axes: dismantling pathological condensates, inducing maladaptive phase transitions, and harnessing artificial condensates as delivery vehicles or therapeutic agents. Each strategy offers distinct advantages but also presents challenges regarding specificity, context dependence, and potential interference with physiological condensates.

### Challenges and translational bottlenecks of LLPS-based therapeutics

Although LLPS-based strategies are conceptually attractive, several features of condensate biology make clinical translation difficult. First, condensates are highly heterogeneous and context-dependent. The same scaffold protein can form distinct condensates with different partners and material states in different tumor types, treatment stages, or microenvironments. As a result, perturbing one “LLPS-prone” factor may have opposite effects depending on cellular context, and it is not yet clear how to predict which condensates will be most relevant for a given therapy. Second, physiological condensates are widespread in normal tissues and often share scaffolds and motifs with pathological assemblies. Agents that broadly disrupt LLPS risk interfering with essential cellular functions, so future drugs will need to recognize specific interaction motifs, PTM states, or client dependencies that are enriched in tumor condensates rather than in normal cells.

A further challenge is how to monitor and control condensates *in vivo*. Most mechanistic data come from fixed cells or *in vitro* reconstitution, while practical pharmacodynamic markers for condensate remodeling in patients are still lacking. It also remains difficult to predict how small molecules partition into, or are buffered by partition into or be buffered within, different condensates; in some settings, drugs may become concentrated in a target condensate, whereas in others they may be sequestered away from critical substrates. Finally, delivery strategies must ensure sufficient local drug levels in the intended condensate without creating unintended “drug sinks” or promoting new adaptive assemblies. These issues highlight the need for closer integration of condensate biology with quantitative imaging, pharmacology, and drug delivery when designing LLPS-centered therapeutic approaches.

## Conclusions

Biomolecular condensates formed via LLPS have redefined our understanding of subcellular organization and regulation in cancer biology. Far from being inert compartments, these dynamic condensates act as versatile hubs that coordinate oncogenic processes, including transcriptional regulation, metabolic reprogramming, DNA damage repair, autophagic flux, and immune evasion. Under chemotherapeutic, radiotherapeutic, or targeted therapy-induced stress, LLPS enables the rapid assembly of adaptive compartments such as SGs and transcriptional condensates, thereby buffering lethal insults, sustaining survival signaling, and promoting treatment resistance.

Despite these insights, the complexity of LLPS in cancer remains incompletely resolved. The biochemical composition, biophysical properties, and dynamic behaviors of condensates vary across cancer types and treatment contexts. For example, condensates formed in response to oxidative stress may differ substantially in content and function from those induced by kinase inhibition. Moreover, many critical biomolecular “nodes” within condensates, including RBPs, intrinsically disordered regions, and scaffold-client interaction networks, are still poorly annotated in the cancer setting.

A deeper understanding of the properties and assembly mechanisms of biomolecular condensates is likely to open new avenues for therapeutic innovation. Strategies aimed at dismantling aberrant condensates or driving irreversible phase transitions of key pathogenic components hold promise for re-sensitizing refractory tumor cells to existing treatments. In parallel, exploiting phase separation to engineer drug-encapsulating droplets or LLPS-guided carriers may enhance intracellular drug concentration and pharmacological efficacy, particularly for poorly soluble agents. In summary, elucidating the principles that govern intracellular phase separation will have broad implications for future drug development and the rational design of therapeutic strategies to overcome cancer therapy resistance.

### Open questions

Several key questions remain to be addressed before condensate biology can be fully integrated into precision oncology.

One central issue is the context-specificity of biomolecular condensates: their composition, material state, and regulatory inputs differ across tumor lineages, subclones, and treatment stages, and it is still unclear which condensate features truly drive therapy resistance rather than simply reflect altered cell states. Another major challenge is the lack of quantitative tools to visualize and monitor condensate remodeling *in vivo*, particularly in patients, where current imaging modalities and biomarkers are insufficient to capture rapid, small-scale changes or drug partitioning within condensates. At a mechanistic level, the causal relationships between LLPS, genetic evolution, epigenetic plasticity, and metabolic reprogramming remain to be defined within a unified framework. Finally, principles for selectively targeting pathological condensates while preserving physiological assemblies are only beginning to emerge.

Addressing these open questions will be essential for translating the conceptual advances summarized in this review into safe and effective LLPS-based interventions for therapy-resistant cancers.

## Acknowledgments

This work was supported by the 10.13039/501100001809National Nature Science Foundation of China project (grant nos. 82373178 and 82073207).

## Author contributions

D.S. and C.W. designed and discussed the content. P.H., H.D., and N.Y. wrote and examined the manuscript. J.Y., S.R., and Y.Q. designed the illustrations. All authors have read and agreed to the published version of the manuscript.

## Declaration of interests

The authors declare no competing interests.
