# Supplementary material for: Liquid-liquid phase separation in cancer therapy resistance
Source: iScience. 2026 Mar 20;29(4):115420. doi: 10.1016/j.isci.2026.115420 (PMC13091035; doi:10.1016/j.isci.2026.115420)
Supplement: Document S1. Tables S1 and S2 [file mmc1.pdf]

iScience, Volume *29*

Supplemental information

Liquid-liquid phase separation in cancer therapy resistance

Peng Hu, Huaze Ding, Ningning Yue, Jianxin Ye, Shengzhe Ruan, Yike Qian, Changping Wang, and Dianwen Song

Table S1. Literature search strategies

Database	Search fields	Search query	Date range	Last search	Notes
PubMed	All fields	("liquid-liquid phase separation" OR LLPS OR "biomolecular condensate" OR "phase separation") AND (cancer OR tumor OR tumour OR neoplasm) AND (therapy OR treatment OR drug OR chemotherapy OR radiotherapy OR immunotherapy OR targeted) AND (resistance OR "drug resistance" OR "therapy resistance" OR chemoresistance OR radioresistance)	Inception to Nov 2025	November 19, 2025	Combined LLPS, cancer, therapy, and resistance terms.
Web of Science Core Collection	Topic (TS)	TS = (("liquid-liquid phase separation" OR LLPS OR "biomolecular condensate*" OR "phase separation") AND (cancer OR tumor OR tumour OR neoplasm) AND (therapy OR treatment OR drug OR immunotherapy OR radiotherapy OR chemotherapy) AND (resistance OR "drug resistance" OR "therapy resistance"))	Inception to Nov 2025	November 19, 2025	TS searches title/abstract/ keywords.

Table S2. Evidence-strength appraisal of key studies

Ref No.	Condensate/ driver	Cancer context/ model	Therapy/resistance context	LLPS evidence	In vivo/clinical support	Evidence tier
43	FOXM1 transcriptional condensates	Breast cancer	Condensate targeting as sensitization strategy (review)	Condensates; LLPS-deficient mutant (S376E); small-molecule disruption (FIP4)	Yes (xenograft; review)	Tier 1
62	CRC TF condensates	Osteosarcoma	Cisplatin & methotrexate resistance	TF condensates	No (not stated in review)	Tier 2
63	FOXP1 transcriptional condensates	Small cell lung cancer	Cisplatin chemoresistance; PARPi combination strategy	Stress-induced phase separation; condensate disruption via ARV-771	Yes (PDX; review)	Tier 1
66	RXRγ–LSD1 transcriptional condensates	Small cell lung cancer	Tumor chemoresistance (study title)	Phase separation of RXRγ reported (study title)	No (not stated in review)	Tier 2
67	FUS-mediated phase separation of glucocorticoid receptor	T-cell lymphoma	Glucocorticoid resistance (study title)	FUS-mediated phase separation reported (study title)	No (not stated in review)	Tier 2
68	Androgen receptor (AR) condensates	Prostate cancer (CRPC)	Antiandrogen resistance (study title)	AR phase separation reported; targeting AR LLPS	No (not stated in review)	Tier 2
81	NSD2-mediated SRC-3 LLPS	Multiple myeloma	Bortezomib response / sensitization (study title)	SRC-3 LLPS driven by NSD2; targeting LLPS sensitizes bortezomib	No (not stated in review)	Tier 2
83	Phospho-HDAC6 nuclear condensates (chromatin architecture)	Triple-negative breast cancer	Drug-tolerant chromatin reprogramming (review); resensitization discussed	Phosphorylation-dependent phase separation (study title/review)	No (not stated in review)	Tier 2
106	NBS1 lactylation	Cancer cells (not	Chemotherapy resistance	Phase-separation/repair-	No (not stated in	Tier 2

	(MRN/DDR regulation)	specified in review)	(study title)	foci model discussed in review; lactylation promotes repair capacity	review)	
107	RNF168 nuclear condensates regulated by SENP1	Colon/colorectal cancer	Drug resistance (study title; review)	RNF168 phase separation; SENP1 dissolves condensates (review)	Clinical association mentioned	Tier 2
108	LINP1 RNA–RNA driven condensates	Cancer cells (not specified in review)	Chemo-/radiotherapy resistance via NHEJ	Self-assembly via RNA–RNA interactions (review)	No (not stated in review)	Tier 2
109	LINP1	Breast cancer	Chemoresistance (study title)	Linked to LLPS/condensate framework in review (see Ref.108)	No (not stated in review)	Tier 2
110	KAT6A nuclear condensates (sequester PARP1)	Ovarian cancer	PARP inhibitor resistance (review/title)	IDR-driven condensates; stabilized by APEX1 (review)	No (not stated in review)	Tier 2
122	Ferritin phase separation driven by lncRNA URB1-AS1	Hepatocellular carcinoma	Sorafenib-induced ferroptosis suppression	Ferritin phase separation reported; reduces labile iron (review/title)	Clinical correlation mentioned	Tier 2
131	p62/SQSTM1–NBR1 condensates	Cancer cells	TKI-induced lysosomal stress	Enhanced LLPS of p62/NBR1 under drug-induced stress	No (not stated in review)	Tier 2
139	Nur77 phase separation increases liquidity of p62 condensates	Cancer cells (not specified)	Celastrol-induced mitophagy (study title)	Phase separation of Nur77; modulates p62 condensate material properties	No (not stated in review)	Tier 2
144	HNRNPH1 droplets induced by PTK6 phosphorylation	Colorectal cancer	Stress survival via autophagy (review); apoptosis suppression	PTK6-driven HNRNPH1 phase separation (study title/review)	No (not stated in review)	Tier 2
162	RNF144A–	Osteosarc	Chemothera	SG	No (not	Tier 2

	VRK2–G3BP1 pathway controlling SG assembly	oma	py resistance (review)	formation/assembly via core SG protein G3BP1 (review/title)	stated in review)	
163	RIOK1-enriched stress granules sequestering PTEN mRNA	Hepatocellular carcinoma	TKI exposure; donafenib resistance discussed in review	RIOK1 phase separation/stress granules (study title/review)	Yes	Tier 1
49	YAP nuclear condensates induced by IFN-γ	Tumor models	Anti-PD-1 immunotherapy resistance	IFN-γ promotes YAP phase separation (study title/review)	No (not detailed in review)	Tier 2
50	KAT8–IRF1 transcriptional condensates	Tumor models	Immune checkpoint resistance	Phase separation of KAT8–IRF1 (study title)	No (not detailed in review)	Tier 2
177	Mutant NF2 phase separation trapping cGAS–STING	NF2-mutant tumors	Immune evasion; reduced type I IFN signaling	Induced phase separation of mutant NF2 (study title)	No (not detailed in review)	Tier 2
184	Enzyme-triggered in situ LLPS of peptides into droplets	A549 xenograft (review)	Synergy with sorafenib	In situ LLPS/self-coacervation into droplets (review/title)	Yes (xenograft; review)	Tier 1
88	IGF2BP1 phase separation-mediated ITGB1 mRNA stabilization	Oral cancer	Cisplatin chemoresistance (study title)	Enhances IGF2BP1 phase separation (study title)	No (not stated in review)	Tier 2

Filling rules (qualitative evidence tiering used for transparency in this narrative review):
Tier 1: Phase separation/condensate evidence plus functional perturbation linked to resistance, with in vivo/clinical support.
Tier 2: Phase separation/condensate evidence plus functional link to resistance, but limited/unspecified in vivo/clinical support.
Tier 3: Primarily associative/correlative evidence; limited mechanistic or functional validation.
